# The effects of lifelong blindness on murine neuroanatomy and gene expression

**DOI:** 10.3389/fnagi.2015.00144

**Published:** 2015-07-24

**Authors:** Charles W. Abbott, Olga O. Kozanian, Kelly J. Huffman

**Affiliations:** ^1^Interdisciplinary Neuroscience Graduate Program, University of California, RiversideRiverside, CA, USA; ^2^Department of Psychology, University of California, RiversideRiverside, CA, USA

**Keywords:** gene expression, intraneocortical connections, LGN, plasticity, vision, aging

## Abstract

Mammalian neocortical development is regulated by neural patterning mechanisms, with distinct sensory and motor areas arising through the process of arealization. This development occurs alongside developing central or peripheral sensory systems. Specifically, the parcellation of neocortex into specific areas of distinct cytoarchitecture, connectivity and function during development is reliant upon both cortically intrinsic mechanisms, such as gene expression, and extrinsic processes, such as input from the sensory receptors. This developmental program shifts from patterning to maintenance as the animal ages and is believed to be active throughout life, where the brain’s organization is stable yet plastic. In this study, we characterize the long-term effects of early removal of visual input via bilateral enucleation at birth. To understand the long-term effects of early blindness we conducted anatomical and molecular assays 18 months after enucleation, near the end of lifespan in the mouse. Bilateral enucleation early in life leads to long-term, stable size reductions of the thalamic lateral geniculate nucleus (LGN) and the primary visual cortex (V1) alongside a increase in individual whisker barrel size. Neocortical gene expression in the aging brain has not been previously identified; we document cortical expression of multiple regionalization genes. Expression patterns of *Ephrin A5, COUP-TFI*, and* RZRβ* and patterns of intraneocortical connectivity (INC) are altered in the neocortices of aging blind mice. Sensory inputs from different modalities during development likely play a major role in the development of cortical areal and thalamic nuclear boundaries. We suggest that early patterning by prenatal retinal activity combined with persistent gene expression within the thalamus and cortex is sufficient to establish and preserve a small but present LGN and V1 into late adulthood.

## Introduction

Functionally distinct sensory and motor regions are generated through patterning of the nervous system during mammalian development. These regions represent discrete subunits of an elaborate network regulating multi-sensory integration, complex motor function and other high-level processes. Much of complex sensory processing and sensori-motor integration takes place within the neocortex, which is subdivided into many discrete, topographically organized primary, secondary and tertiary sensory and motor areas. Information from sensory receptors (such as those found in the cochlea, skin and retina) are relayed to the neocortex via thalamocortical afferents (TCAs) from distinct thalamic nuclei. This input is processed in a modality-specific manner, ultimately resulting in perception. Exactly how and why the brain ages is not fully understood, but most agree that a decline in neurotransmitter function, generation of reactive oxygen species, dysregulation of calcium signaling, and mitochondrial dysfunction are involved in the maladaptive features of brain aging (Mattson et al., 2002; Burke and Barnes, 2006; Toescu and Verkhratsky, 2007).

Genetic and anatomical manipulations to the neocortical network have both been employed extensively to uncover developmental mechanisms that drive arealization of the neocortex (Assimacopoulos et al., 2012; Erzurumlu and Gaspar, 2012). Two distinct hypotheses have been proposed to account for early developmental patterning of the neocortex: the Protomap and Protocortex Hypotheses (Rakic, 1988; O’Leary, 1989). The Protomap Hypothesis describes the role of cortically intrinsic features, such as morphogens and transcription factors like Wnt and Fgf family proteins in cortical patterning (Donoghue and Rakic, 1999; Garel et al., 2003; Huffman et al., 2004), whereas the Protocortex model emphasizes the tabula rasa-like character of the early cortical progenitors and posits that sensory area identity is determined from sensory input from the periphery (Schlaggar and O’Leary, 1991; Nakagawa et al., 1999). Once at odds, scientists now concur the validity of both models of early neocortical development and patterning. However, neither models addresses how neural gene expression and sensory experience interact throughout life, providing maintenance of functionality as well as plasticity. Thus, a goal of this study is to untangle how cortically extrinsic processes, such as those related to sensory input, and cortically intrinsic processes, such as gene expression, influence each other throughout life. To do this, we reweighted the sensory input to the cortex by eliminating all input from the eyes and examined neuroanatomy and gene expression in the brains of aging mice.

Manipulation of visual input has long been used to assess deprivation-induced plasticity within the neocortex of cats, primates and more recently, rodents (Wiesel and Hubel, 1963; Hubel and Wiesel, 1964; Kaas et al., 1990; Arckens et al., 1998, 2000; Sawtell et al., 2003; Frenkel and Bear, 2004; Tagawa et al., 2005; Hofer et al., 2006; Cnops et al., 2007; Lehmann and Löwel, 2008; Van Brussel et al., 2011). Bilateral enucleation, specifically, has been used extensively, and to great effect, revealing a role for retinal input in dendritic spine formation, areal border maintenance, intraneocortical connection (INC) development and proper patterning of gene expression (Karlen et al., 2006; Karlen and Krubitzer, 2009; Bock et al., 2010; Desgent et al., 2010; Dye et al., 2012).

Although great strides have been made in our understanding of how patterning progresses during early development, far less is known about maintenance throughout the lifespan of the animal, particularly during senescence. Understanding the intrinsic molecular mechanisms and extrinsic sensory cues that shape aging-related plasticity is of utmost importance. In an effort to further explore this question, the goals of the project were two fold: (1) to evaluate the effects of long term visual deprivation on INCs and areal/nuclear organization in the aging brain using anatomical tracing methods and cytochrome oxidase (CO) staining; (2) to determine how a major change in sensory input early in life would alter gene expression of cortical patterning genes in old age. The five genes studied include: *Cadherin 8* (*Cad8*), which is implicated in the division of neuronal circuits via its differential expression patterns (Suzuki et al., 1997); *COUP-TFI*, a transcription factor which is critical for regulation of sensory and frontal/motor patterning in the cortex (Armentano et al., 2007; Faedo et al., 2008); *Ephrin A5*, an axonal guidance cue which organizes topographical patterning, and is expressed highly in the putative somatosensory cortex (Mackarehtschian et al., 1999; Vanderhaeghen et al., 2000; Dye et al., 2011a,b); *LIM homeobox protein 2* (*Lhx2*), which specifies regional fate in cortical progenitors (Chou et al., 2009); and *retinoic acid receptor related orphan beta* (*RZRβ* or *ROR*β), a marker for primary sensory areas which cell-intrinsically drives cytoarchitectural patterning of layer IV neurons during development of barrels in the somatosensory cortex (Nakagawa and O’Leary, 2003; Jabaudon et al., 2012).

**Figure 1:**
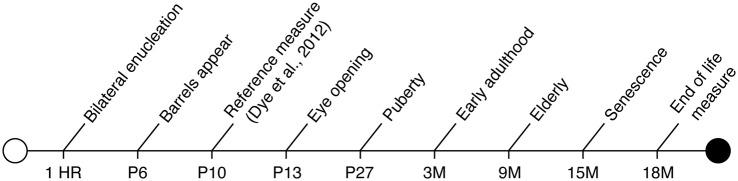
**Timeline of murine developmental milestones from birth through death including definitive timepoints during early development, sexual maturation and aging**.

To accomplish these primary goals, we conducted bilateral enucleation in newborn mice, thus removing all visual input to the developing brain. Our blind mice were previously analyzed at postnatal day (P) 10 (10 days after enucleation; Dye et al., 2012), and this study extends the previous work to a long-term experiment in the aged, blind animal (see Figure 1 for timeline).

Our results demonstrate that bilateral enucleation in early development induces lifelong changes in cortical connectivity, specifically at the somatosensory-visual (S-V) areal boundary, areal, barrel and nuclear size as well as changes in gene expression. Here we will discuss how elimination of sensory input from a single system throughout life can impact anatomical features and gene expression in the brain of the aging animal.

## Materials and Methods

### Mouse Colony

All procedures were conducted in accordance with protocol guidelines approved by the Institutional Animal Care and Use Committee (IACUC) at the University of California, Riverside. CD-1 mice, originally obtained from Charles River Laboratories, were bred in an animal facility at UCR and aged to 18 months after bilateral enucleation on the day of birth. All animals were housed under a standard 12 h/12 h light/dark cycle with *ad libitum* access to food and water.

### Newborn Bilateral Enucleation and Aging of Enucleated Mice

As in previous studies (Dye et al., 2012), all animals were enucleated at P0, after being allowed to nurse shortly (~1 h) before surgery. Surgical procedures were conducted within a strict 2 h timeframe. Pups were anesthetized with ketamine (40 mg/kg) and xylazine (5 mg/kg), administered via intraperitoneal injection, and placed briefly on ice before eye removal. A toe-pinch was used to verify surgical level of anesthesia. Next, a small incision was made to open the eyelid. The eye and optic nerve were then freed from all surrounding musculature and removed. Following removal of the eye, the incision was sealed with 0.5–1.0 μL of tissue adhesive (Surgi-Lock instant liquid tissue adhesive, Fisher Scientific, Pittsburgh, PA, USA). Pups were then revived by partially immersing them for 30 s in a lukewarm water bath. The incisions were then coated with Lidocaine hydrochloride jelly USP, 2% (Akorn, Lake Forest, IL, USA) and erythromycin ophthalmic ointment USP, 0.5% (Bausch and Lomb, Rochester, NY, USA) to minimize pain and infection. Anesthesia and revival procedures as outlined above were also conducted on control pups and treated as shams.

### Tissue Preparation

All experimental and control mice were euthanized with a lethal dose of sodium pentobarbital (100 mg/kg), and perfused with 4% paraformaldehyde (PFA) in 0.1 M phosphate buffer (pH 7.4) at 18 months of age. Brains were then extracted and hemisected, with one hemisphere used for *in situ* RNA hybridization, and the other used for postmortem dye tracing. Hemispheres used for *in situ* RNA hybridization were post-fixed overnight at 4°C, then dehydrated through graded methanol steps before storage in 100% methanol at −20°C. Brain hemispheres reserved for postmortem dye tracing were retained in 4% PFA at room temperature for the duration of tracer transport following dye placement.

### Anatomical Tracing

To visualize ipsilateral INC patterns in control and bilaterally enucleated mice, single DiA and DiI (Invitrogen, San Diego, CA, USA) crystals were placed in postmortem neocortical tissue (*N* = 7 for control and experimental mice). This technique has been used previously to identify mouse INCs in many prenatal and postnatal ages (Dye et al., 2011a,b, 2012). To ensure inter-case reliability dye crystals were placed in one of two locations identified using a coordinate grid: parietal lobe, within primary somatosensory (S1) cortex or the occipital lobe, within an area corresponding with the location of V1. Upon dye placement location (DPL) confirmation, crystals were inserted perpendicular to the cortical surface to a depth of approximately 80 μm. Full dye placement methodology has been described previously (Huffman et al., 2004; Dye et al., 2011a,b, 2012). Following dye placement, hemispheres were stored at room temperature in 4% PFA for a period of 6–8 weeks to allow for transport, and processed further following confirmation of retrograde labeling of thalamic nuclei. Brain hemispheres were then embedded in low-melting point agarose and sectioned at 100 μm in the coronal plane on a vibratome, counter-stained with 4′, 6-diamidine-2-phenylindole dihydrochloride (DAPI; Roche), mounted on glass slides and coverslipped with Vectashield mounting medium for fluorescence (Vector Laboratories, Inc., Burlingame, CA, USA), then imaged using a Zeiss Axio Imager Upright Microscope equipped with fluorescence. Three filters were used to visualize dye: red for DiI, green for DiA and blue for DAPI counterstain labeling (*Excitation wavelengths*-red: Cyanine 3,550 nm; green: green flurescent protein (GFP), 470 nm; blue: DAPI, 359 nm. *Emission wavelengths*-red: Cyanine 3, 570 nm; green: GFP, 509 nm; blue: DAPI, 461 nm). During analysis, the experimenter was blind to condition. Quantification of the positional shift of somatosensory INCs at the S-V boundary was achieved by using an electronic micrometer to measure from the cortical midline to the position of the most medial labeled cell resulting from a somatosensory dye placement.

### Gene Expression Assays

Gene expression assays were carried out following standard protocols for non-radioactive free-floating *in situ* RNA hybridization (*N* = 6 for control and experimental animals; Huffman et al., 2004; Dye et al., 2011a,b, 2012; El Shawa et al., 2013). The following probes were used to identify patterns of gene expression at mice aged to 18 months: *Ephrin A5, Cadherin 8* (*Cad8*, a gift from Masatoshi Takeichi, Riken Center for Developmental Biology, Japan), *nuclear receptor subfamily 2, group F, member 1* (*COUP-TFI*), *RZRβ* (both gifts from John Rubenstein, UCSF) and *Lhx2* (a gift from Juan Botas, Baylor College of Medicine). In preparation for *in situ* RNA hybridization, brain hemispheres were rehydrated through a methanol series, embedded in gelatin-albumin together with a positive control (wild-type P0 brain), then sectioned on a vibratome in the coronal plane at a thickness of 100 μm. Following hybridization all sections were mounted in glycerol on glass slides, coverslipped and digitally imaged in high resolution using a Zeiss Axio camera fitted to a Zeiss Stereo Discovery V12 stereomicroscope, using Axiovision software. Following *in situ* hybridization (ISH), neocortical gene transcript density was analyzed within specific regions of interest (ROI) using ImageJ, with values presented as area fraction of total ROI. During analysis, the experimenter was blind to condition. Full methods have been described previously (Dye et al., 2012; El Shawa et al., 2013).

### Cytochrome Oxidase Staining

CO staining was used to visualize thalamic nuclei, V1 and the barrels in somatosensory cortex in control and enucleated animals (*N* = 5). Hemispheres from 18 month old enucleated and control mice were cryoprotected post-perfusion with a solution of 30% sucrose in phosphate buffer for 48 h at 4°C, then cryosectioned at 40 μm in the coronal plane. Sections were then reacted for CO at 37°C, mounted on glass slides, coverslipped and imaged in high resolution using a Zeiss Axio camera fitted to a Zeiss Stereo Discovery V12 stereomicroscope, using Axiovision software. ImageJ was used to electronically measure the size of dorsal lateral geniculate nucleus (dLGN), V1 and individual barrels in somatosensory cortex, with data displayed as change from baseline (control) measure. During analysis, the experimenter was blind to condition.

### Cell Nuclei Counting and Analysis

Cell nuclei were counted in the V1 and dorsal lateral geniculate nucleus (dLGN) to quantify differences in cell packing density. Brightfield Z-stack images of 30 μm thick Nissl-stained coronal sections were collected then analyzed using the ITCN plugin for ImageJ. First an electronic ROI was drawn over the selected region, then area and cell nuclei number were quantified. Volume measurements were made by calculating the distance between the top and bottom sections of the stack and multiplying by ROI area.

### Statistical Analyses

Significance was established using two sample *t*-tests assuming equal variance among samples. Data are presented as mean ± s.e.m.

## Results

We have previously reported alteration in both neocortical gene expression and INCs in mice at P10, following early bilateral enucleation on the day of birth (Dye et al., 2012). Specifically, we observed a positional shift in expression of *ephrin A5* and a corollary shift in INC projections at the somatosensory-visual border, highlighting short-term plasticity that occurred before natural eye opening. Building on these initial findings in young animals the present report seeks to determine the range of effects of early bilateral enucleation on the anatomical, connectional and gene expression patterns in the aging mouse.

### Long-Term Thalamic Nuclei Size Alterations in Response to Bilateral Enucleation

**Figure 2:**
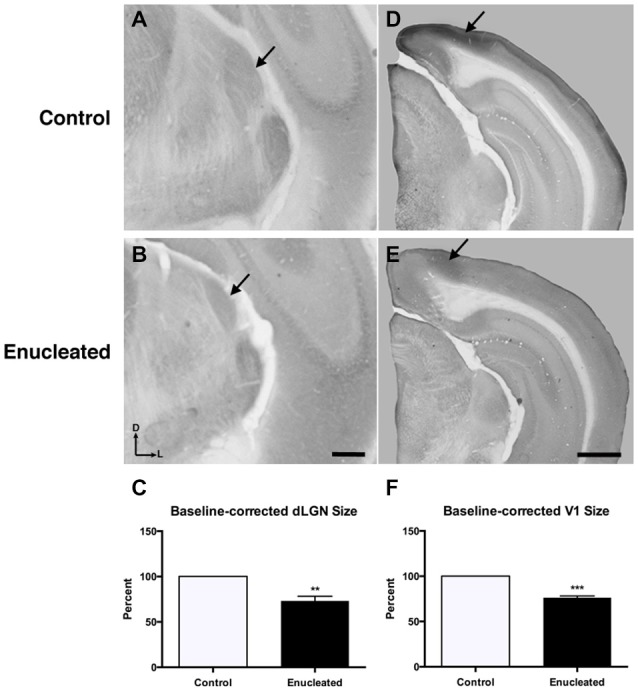
**Reduced dLGN and V1 18 months post newborn bilateral enucleation**. Cytochrome oxidase staining was used to reveal dLGN architecture and mark primary visual cortex in 18 month control and bilaterally enucleated brains. **(A,B)**: Panels are high magnification views of the dLGN (arrows) and surrounding areas of the dorsal thalamus after sectioning at 40 μm in the coronal plane. CO staining indicates that visual thalamic nuclei (dLGN) are present, but reduced in enucleated brains **(B)** as compared to controls **(A)**. **(C)**: The dLGN size in enucleated brains is significantly reduced (73.13 ± 5.192%) compared to control brains. **(D,E)**: Panels show hemisected brains sectioned at 40 μm in the coronal plane. A reduction, but not elimination of primary visual cortex (arrows), is observed in enucleated tissue compared to that of controls. **(F)**: V1 size in enucleated cortex is significantly reduced (76.35 ± 1.850%) compared to controls. Tissue oriented with dorsal up and lateral to the right. dLGN Scale bar = 500 μm. dLGN: dorsal lateral geniculate nucleus. **P < 0.01; ***P < 0.001. V1 Scale bar = 500 μm. *N* = 5 per condition.

**Figure 3:**
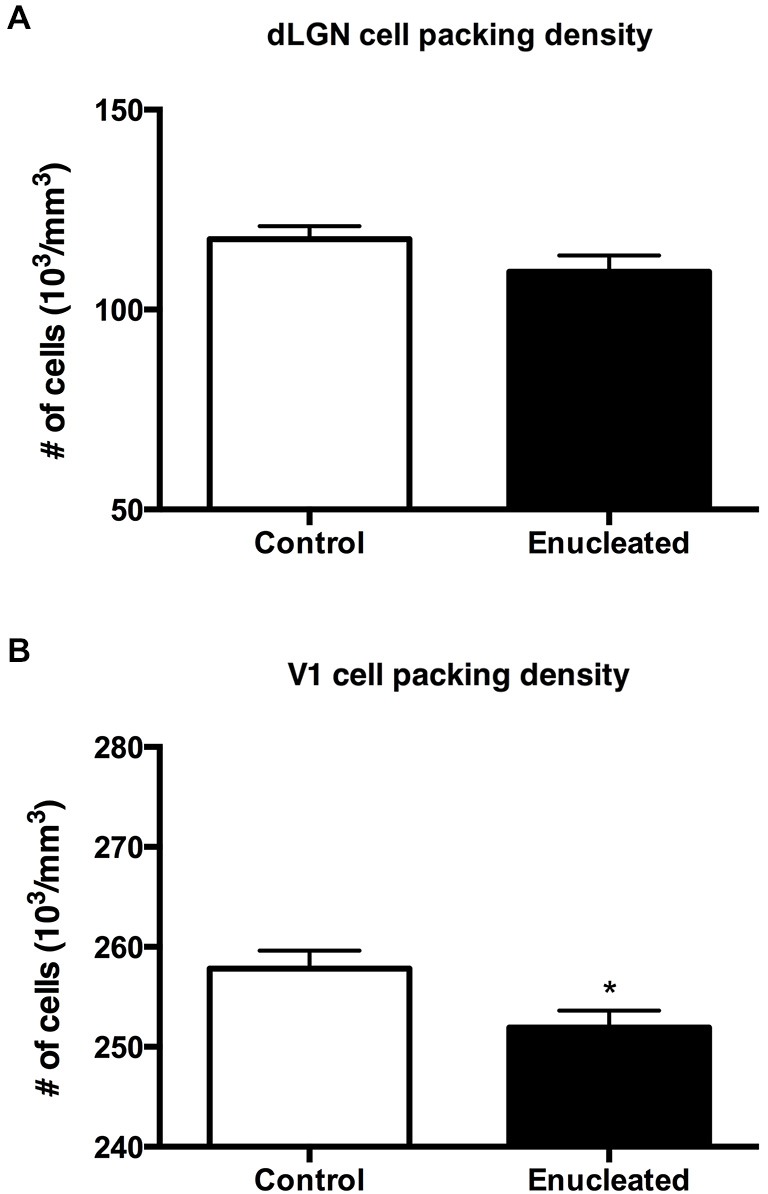
**Summary cell density graphs**. Graphs showing packing density (expressed as cells × 10^3^) of cell nuclei from coronal sections in V1 and dLGN. **(A)** Cell packing density within the dLGN of enucleated mice does not significantly differ from that of controls (control 117.6 ± 3.206; enucleated 109.5 ± 4.065; *P* > 0.05). **(B)** Cell packing density within V1 of enucleated mice is significantly reduced when compared to controls (control 257 ± 1.793; enucleated 251.9 ± 1.714; *P < 0.05).

Initial work for this study began with an investigation of the effects of early bilateral enucleation on the long-term maintenance of the dLGN using CO staining techniques (Figures 2A,B; arrows). Elderly, 18 month old enucleated mice display a significant reduction in dLGN when compared to control animals (Figure 2C, 26.87 ± 5.192% reduction from control; *N* = 5, *P* < 0.01) without a change in cell packing density (Figure 3A: control 117.6 ± 3.206; enucleated 109.5 ± 4.065; *P* > 0.05). These results extend our previous finding of a reduction in dLGN size by P10 (Dye et al., 2012) by demonstrating that the small size of the dLGN is maintained throughout life. Examination of the genes *Ephrin A5, Cad8*, *COUP-TF1, Lhx2* and *RZRβ* at this elderly time-point revealed no appreciable expression in the dLGN, vLGN or VP in either the control or enucleated cases (data not shown).

Reduced thalamic volume, particularly in the dLGN, has been previously reported in studies examining blind animals ranging from early postnatal time-points to P180 or 6 months (Heumann and Rabinowicz, 1980; Warton et al., 1988; Asanuma and Stanfield, 1990; Dehay et al., 1996; Williams et al., 2002; Dye et al., 2012), however studies lasting into senescence in a blind mouse model have not been done previously. This report demonstrates a stabilized reduction in dLGN size that persists into terminal stages of the murine lifespan (Figures 2A–C).

### Bilateral Enucleation Reduces Size of Primary Visual Cortex

Reacting tissue for CO activity revealed a significant reduction in size of and cell packing density within the V1 (arrows) in elderly, enucleated animals when compared to controls (Figures 2D–F; 23.65 ± 1.850% reduction from control; *P* < 0.001; *N* = 5; Figure 3B: control 257 ± 1.793; enucleated 251.9 ± 1.714; *P* < 0.05). Furthermore, cells in V1 exhibited a stark reduction in CO activity, and a loss of layer-specific CO activity patterns: control mice exhibit strong activity in layers 2–4 of V1 (Figure 2D, arrow) whereas enucleated animals displayed a uniform, and reduced, CO activity gradient across all cortical layers (Figure 2E, arrow).

### Bilateral Enucleation Results in Larger Barrels Within Somatosensory Cortex of Aging Mice

**Figure 4:**
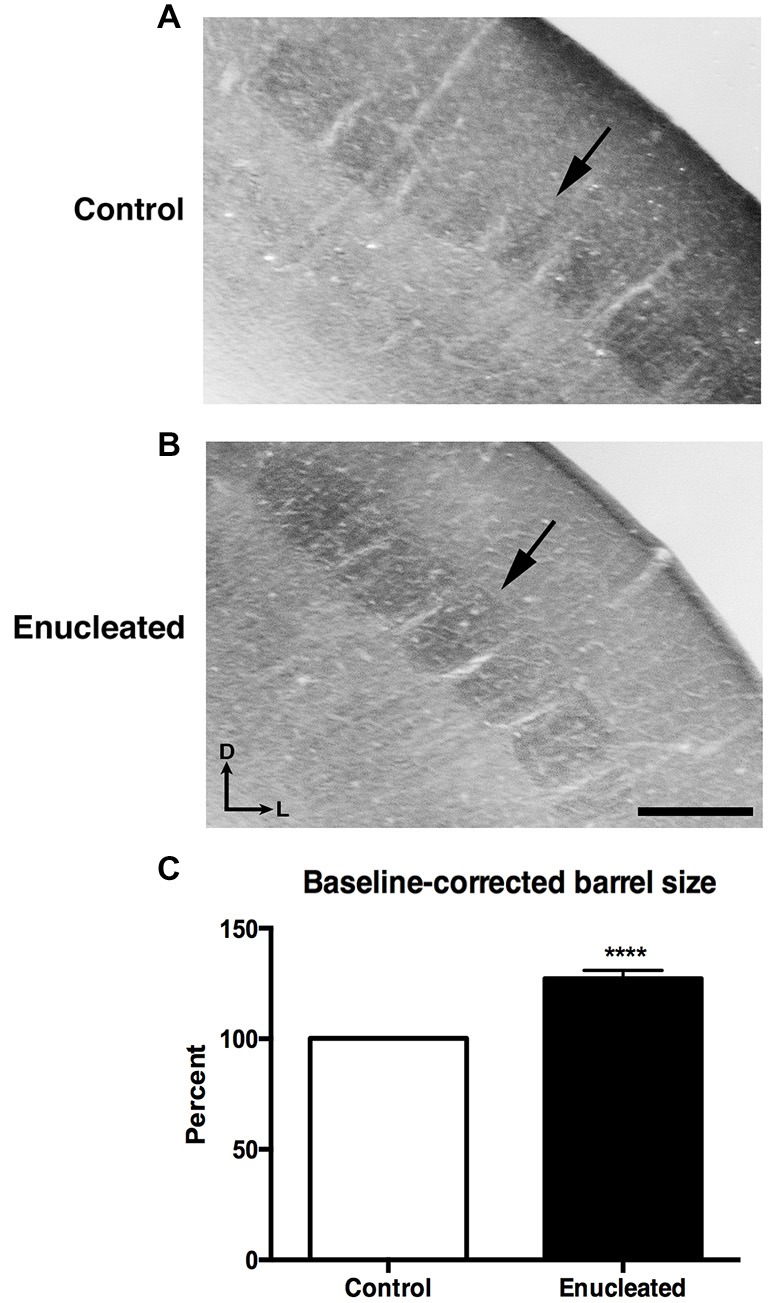
**Increased barrel size 18 months post bilateral enucleation at birth**. Cytochrome oxidase (CO) staining was used to reveal barrel size in control and bilaterally enucleated somatosensory cortex. **(A,B)**: Panels are high magnification views of barrels and surrounding areas of somatosensory cortex after sections at 40 μm in the coronal plane. CO staining shows increased barrel size in enucleated brains **(B)** as compared to controls **(A)**. **(C)**: Barrel size in enucleated brains is significantly increased (127.2 ± 3.692%) compared to control brains. Tissue oriented with dorsal up and lateral to the right. Scale Bar = 200 μm, ****P < 0.0001.

In sections stained for CO, we observed a very clear barrel field (Figures 4A,B), with barrels (arrows) corresponding to individual whisker pads on the face of the mouse, in both control and enucleated aging mice. Upon measurement of individual barrels, a clear increase in size of individual barrels was observed in the brains of enucleated mice (Figure 4C: 127.2 ± 3.692% increase from control, *P* < 0.0001). This is an example of cross-model plasticity that occurs after the loss of a single sensory system.

### Gene Expression in Late Adulthood Following Early Bilateral Enucleation

**Figure 5:**
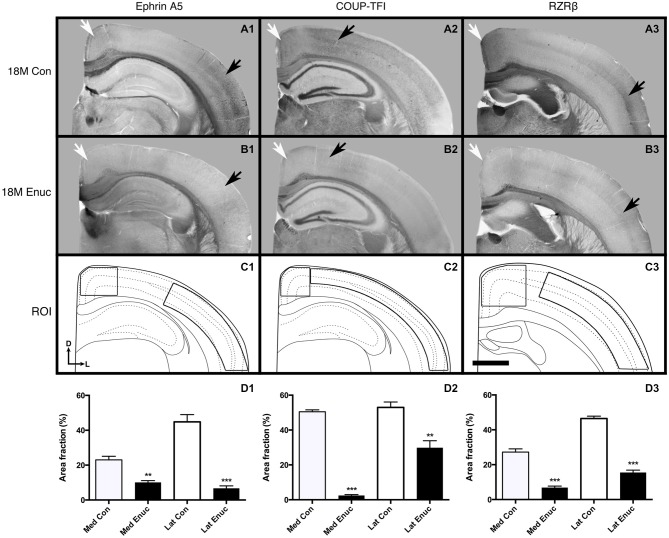
**Gene expression in 18 month old control and enucleated mice**. *In situ* RNA hybridization was used to determine the distribution of transcripts for *ephrin A5* **(A1–B1)**, *COUP-TF1* **(A2–B2)**, and RZRβ **(A3–B3)** in 18 month control and enucleated brains as labeled. All panels **(A1–B3)** are low magnification views of one representative hemisphere after sectioning at 100 μm in the coronal plane for each gene. *Ephrin A5* expression in 18 month old control **(A1)** animals was primarily observed in layers 2/3 and 5 of caudolateral cortex and near the midline in the rostromedial portion of visual cortex (arrows). Enucleation at birth showed a significant reduction in *Ephrin A5* cortical expression 18 months later (compare **A1** to **B1** arrows). *COUP-TF1* is present in 18 month old control and enucleated (**A2,B2**, arrows) parietal cortex, with significantly lower expression levels in these areas in 18 month old enucleated brains compared to controls. Robust expression of *RZRβ* was observed in rostromedial cortex and layers 4/5 of parietal cortex of control (**A3** arrows) brains, with significantly reduced *RZRβ* transcript levels in enucleated brains (**B3** arrows). **(C1–C3)**: Cartoon illustration of respective cortical regions of interest overlaid on a representative coronal section. **(D1)**: Percent area fraction of cells expressing *ephrin A5* within ROIs located within medial and lateral cortex **(C1)**. A sharp decrease in transcript density is observed in enucleated cases (Lateral: 44.81 ± 4.151%; Medial: 10.14 ± 0.9747%) when compared to control animals (Lateral: 6.720 ± 1.413%; Medial: 23.02 ± 2.031%; *P* < 0.001, *P* < 0.01) **(D2)**: Percent area fraction of cells expressing *COUP-TF1* within ROIs located in parietal cortex **(C2)**. Percentage of *COUP-TF1* transcripts of enucleated animals (lateral: 29.89 ± 3.963%; medial: 2.451 ± 0.5394) were significantly different from controls (lateral: 53.00 ± 3.084%; medial: 50.48 ± 1.073; *P* < 0.01, *P* < 0.001). **(D3)**: Percent area fraction of cells expression *RZRβ* transcripts within ROIs located in rostral parietal cortex **(C3)**. Percentage of *RZRβ* transcripts in enucleated brains (lateral: 15.48 ± 1.351%; medial: 6.868 ± 0.8560) were significantly lower than controls (lateral: 46.47 ± 1.356%; medial: 27.22 ± 1.836; *P* < 0.001). All sections are oriented with dorsal (D) up and lateral (L) to the right. *N* = 6 for each condition. **P < 0.01; ***P < 0.001. Scale bar = 1000 μm.

Two out of five genes examined were not observable in the neocortex of 18 month old control and enucleated brains, including; *Cad8* and *Lhx2* (data not shown). As the expression patterns of these genes have not been examined in senescence, it is very possible that this is a direct effect of aging. Robust* ephrin A5* gene expression was present in rostral-medial visual cortex (Figure 5, **A1** white arrow) and in layers 2/3 and layer 5 of parietal cortex in control animals (Figure 5, **A1** black arrow). In comparison, there was very limited neocortical expression patterns present in 18 month enucleates (Figure 5, **B1** arrows). Analysis of transcript density within a medial cortical ROI corresponding with the S-V boundary revealed significantly reduced expression of *ephrin **A5*** (Figure 5 **D1**, Control 23.02 ± 2.031%, enucleated 10.14 ± 0.9747%; *P* < 0.01). Analysis of lateral neocortical *ephrin A5* gene expression revealed significantly higher transcript levels in controls (Figure 5, **D1**; 6.720 ± 1.413%, *P* < 0.001), when compared to enucleated brains (Figure 5, **D1**; 44.81 ± 4.151%).

Neocortical* COUP-TFI* expression, was light, but detectable in neocortex of both control and enucleated mice (Figure 5, **A2** and **B2** arrows). However, analysis of transcript density within a medial cortical ROI corresponding with the S-V boundary revealed significantly reduced expression of *COUP-TFI* (Figure 5 **D2**, Control 50.48 ± 1.073%, enucleated 2.451 ± 0.5394%; *P* < 0.001). Enucleated brains also had significantly reduced transcript density (Figure 5, **D2**; 29.89 ± 3.963%; *P* < 0.01) present in the lateral cortex when compared to controls (Figure 5, **D2**; 53.00 ± 3.084%).

In control brains, *RZRβ* is strongly expressed at the midline and in layer 4 of somatosensory cortex (Figure 5, **A3**, white and black arrows, respectively). In enucleated tissue, *RZRβ* expression is reduced in both locations (Figure 5, **B3** arrows). Analysis of transcript density within a medial cortical ROI corresponding with the S-V boundary revealed significantly reduced expression of* RZRβ* in enucleated animals as compared to controls (Figure 5, **D3**, Control 27.22 ± 1.836%, enucleated 6.868 ± 0.8560%; *P* < 0.001). *RZRβ* expression in enucleated brains was present more laterally, within somatosensory cortex in layer 4 (Figure 5, **B3** black arrow), but notably reduced in this lateral ROI (Figure 5, **D3**; 15.48 ± 1.351%) when compared to control animals (Figure 5, **D3**; 46.47 ± 1.356%; *P* < 0.001. *Ephrin A5, COUP-TF1* and *RZRβ* transcripts were present within a number of large fiber tracts, including the corpus callosum (Figure 5, **A1–B1, A2–B2, A3–B3**), with no observed significant difference between control and enucleated cases.

### Ectopic Ipsilateral INCs and Their Co-Registration with Atypical Gene Expression in Cortex of Enucleated Mice

**Figure 6:**
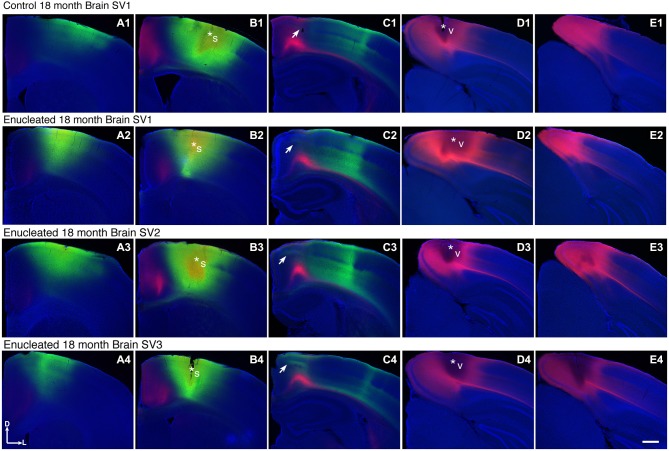
**Ectopic INCs 18 months after early enucleation**. Rostral (left) to caudal (right) series of 100 μm coronal sections of 18 month old hemispheres following placement of DiA or DiI crystals in visual or somatosensory cortices to label INCs **(A1–E4)**. In a representative control brain, the retrogradely labeled cells from a DPL in the parietal somatosensory cortex is found rostral and caudal to the DPL (green labeling in **A1–C1**) and does not overlap with cells or axons labeled by an occipital visual cortex DPL (red labeling in **C1**). The arrow in **(C1)** indicates region of non-overlap between the labeling fields of the two areas, which represents the rostromedial S-V areal boundary in 18 month old mice. In brains from enucleated mice, retrogradely labeled cells from somatosensory DPL are found rostral and caudal to the DPL (green labeling in **A2–C2, A3–C3, A4–C4**). Labeled cells are also found in more medial, ectopic positions not seen in controls, resulting in an overlap with cells labeled by a visual DPL (regions at and below arrows in **C2,–C4**). Dorsal up, medial to the left. Scale bars = 1000 μm. *N* = 7. Section distance from bregma, by column: **(A)**, 1.10 mm; **(B)**, 0.02 mm; **(C)**, -1.58 mm; **(D)**, -3.08 mm; **(E)**, -3.88 mm. Asterisks indicate DPL. S1 DPL: perpendicular to cortical plane centered on 0.02 mm anterior and 2.00 mm lateral to bregma; V1 DPL: perpendicular to cortical plane centered on 3.08 mm posterior and 2.00 mm lateral to bregma. DPL: dye placement location; INC: ipsilateral intraneocortical connection; s: somatosensory areas; S-V: somatosensory-visual; v: visual cortex.

**Figure 7:**
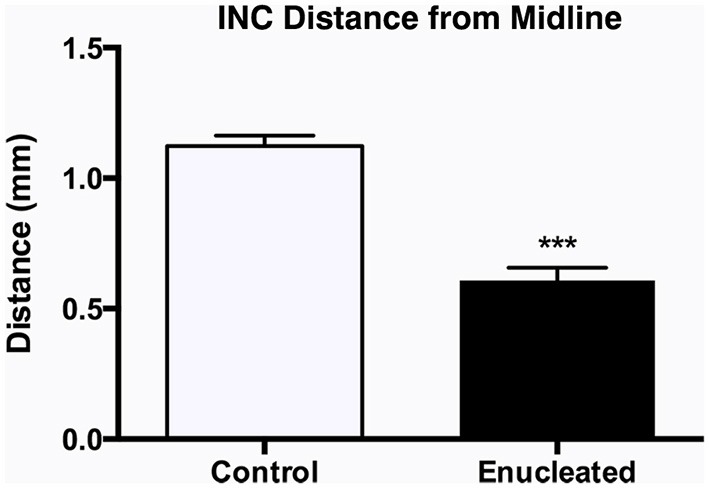
**Quantification of somatosensory INC labeling in the medial parietal cortex of control and enucleated brains**. The distance from the midline edge of the neocortex to the most medial retrogradely labeled cell was measured using a micrometer in image J. Enucleated animals showed a distinct extension of labeling from the somatosensory cortex into what would normally be considered visual cortex labeling, resulting in a reduced distance to midline (0.61 ± 0.05 mm; *N* = 7) when compared to controls (1.12 ± 0.04 mm; *N* = 7). INC: ipsilateral intraneocortical connection. ***P < 0.001.

We have previously characterized ectopic INCs in enucleated mice at P10 (Dye et al., 2012), thus a central goal of this study was to determine if these alterations were maintained, and to what degree, through senescence. To determine the impact of early enucleation on end-of-life cortical connectivity, we used DiI and DiA crystal placements in control and enucleated mice brains to trace INCs. In control mice, retrogradely labeled cells from a DiA DPL in the primary somatosensory (S1) cortex were observed in positions both rostral and caudal to the DPL (Figures 6A–C; green, DPL starred in **B1**). DiI crystals placed in the V1 of control animals resulted in labeling of cells in positions rostral and caudal to the DPL, with dye transport extending from medial parietal cortex to caudal occipital (Figures 6B–E; red, DPL starred in **D1**). In all control cases a clearly defined border was observed between somatosensory and visual areas, with no overlap of labeled cells (Figure 6, **C1**; arrow). In 18 month old mice enucleated at birth DiA crystals placed in S1 resulted in labeled cells both rostral and caudal to the DPL (Figure 6, **A2–C2, A3–C3, A4–C4**, DPLs starred in **B2–B4**) and revealed an extensive expansion of somatosensory DPL labeled cells into tissue normally assigned to the rostromedial section of visual cortex (Figure 6, **C2–C4**; arrows). INC tracing from a DiI DPL in V1 of enucleates resulted in labeling of cells in positions caudal to the DPL (Figure 6, **E2–E4**), and extended rostrally to medial parietal cortex (Figure 6, **B2–B4, C2–C4**; DPLs starred in **D2–D4**) in a manner similar to controls (Figure 6 **B1–E1**). The border between visual and somatosensory cortex (arrows in Figure 6, **C1–C4**) becomes less evident as cells labeled with DiA (from the S1 DPL) are ectopically located in an area that would correspond to visual cortex in a sighted mouse (Figure 6, compare green dye label in **C1** with **C2–C4**). The atypical extension of labeled cells from somatosensory DPLs projects significantly further toward the midline in enucleated animals (Figure 7; distance from midline: control, 1.123 ± 0.04006 mm, *N* = 7; enucleated, 0.6083 ± 0.04843 mm, *N* = 4; *P* < 0.001).

**Figure 8:**
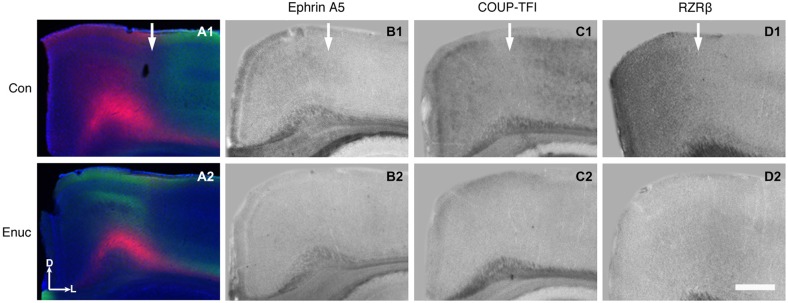
**Abnormal S-V border and diminished gene expression in the rostromedial cortex of 18 month enucleated mice**. High magnification coronal views of 100 μm hemisections of INC tracing (control: **A1**; enucleated **A2**) and ISH (control: **B1–D1**; enucleated: **B2–D2**). Expression of *Ephrin A5*, *COUP-TFI, and RZRβ* in the medial parietal cortex co-registers with the S-V boundary seen in control INCs (Arrows in **A1–D1**). *Ephrin A5*, *COUP-TFI, and RZRβ* expression is reduced or absent in this cortical area in enucleated brains (compare **B1** with **B2**, **C1** with **C2**, and **D1** with **D2**). The area where blindness-induced reduction of gene expression is seen correlated with the shifted S-V border (compare **A2** with **B2–D2**). All sections are oriented with dorsal (D) up and lateral (L) to the right. *N* = 6 per condition. Scale bar = 1000 μm.

In control mice, the S-V area border, located within the rostromedial visual cortex region, is present within *RZRβ, ephrin A5* and *COUP-TFI*—positive zones. For example, the S-V border (Figure 8, **A1** arrow) co-registers with borders of positive expression of *RZRβ, ephrin A5* and* COUP-TFI* (Figure 8, **B1, C1, D1**, arrows). However, in the enucleated mouse, where the S-V border is abnormal and perhaps even absent, there is a clear paucity of gene expression within the area. Specifically, the S-V border is not overtly present in the rostromedial cortex (Figure 8, **A2**) and the specificity of gene expression in this location is gone (Figure 8, **B2, C2, D2**). It has been hypothesized that gene expression may aid in the maintenance of cortical area boundaries throughout life and these data are consistent with this notion (Huffman, 2012).

## Discussion

Studies focused on understanding the complex mechanisms underlying cortical development and arealization have revealed a number of key processes that guide proper formation of adult sensory and motor areas. We have demonstrated that precise developmental targeting of sensory INCs at P0 is controlled, at least in part, by gene expression (Huffman et al., 2004). Complementing these findings we conducted a large-scale study in the CD-1 mouse strain cataloging ipsilateral INC connections and gene expression patterns from embryonic ages to early adulthood (Dye et al., 2011a,b). In this comprehensive study, we described how expression patterns of seven genes previously identified as important for cortical development were correlated in position and timing with patterns of INC. To better understand how sensory input and gene expression interact to generate cortical subdivisions and boundaries, we conducted bilateral enucleation experiments in CD-1 mice at birth (P0), and examined changes in area border formation, connectivity and gene expression 10 days later (Dye et al., 2012). In that report, we identified an atypical extension of *ephrin A5* expression at the S-V border, which correlated positionally with ectopic INCs projecting from somatosensory cortex into the rostro-medial parietal aspect of visual cortex, indicating a role for extracortical input in the establishment of proper areal boundaries. The results from the present study expand the earlier work in the P10 blind mouse where we examine the long-term effects of newborn bilateral enucleation on INCs and gene expression in senescence. The new findings in the aging model lie with reduction of cortical gene expression in the blind, aging mice. In P10 mice, we found ectopic expression that correlated with an extension of cell labeling, suggesting that a potential shift in gene expression was playing a role in the development of the ectopic connections (Dye et al., 2012). However, late in life, after the connections are well past their development, we found that the elimination of visual input effected global and additional layer specific gene expression levels in cortex not found in P10 enucleates. These include reduced expression of *Ephrin A5, COUP-TFI, and RZRβ* in rostro-medial visual and somatosensory cortices, with a distinct reduction at the S-V boundary, despite the persistent phenotype in the INCs. Consistent with our findings at P10, several other genes, including *Cad8* and *Lhx2* did not show any appreciable differences in cortical expression between enucleates and controls.

Studies on genetics of the aging brain have largely focused on microarray data and identified an increase in both global and tissue-specific molecules within the neocortex that function in immune and stress response, and a global decrease in transcription of growth and trophic factors (Lee et al., 2000). Following this work, a handful of other groups have identified differential expression of genes including those involved in neuronal structure, signaling, inflammation and vesicular transport (Jiang et al., 2001; Prolla, 2002; Zahn et al., 2007). Beyond these findings, there is a paucity of data documenting positional information, particularly of genes associated with areal boundaries at earlier postnatal ages, or in animals specifically lacking visual input. In the present study, we identify persistent activity-dependent and independent alterations to the neuroanatomy and connectional patterns of mice at terminal stages of life. By eliminating all retinal input to the thalamus and cortex at birth, we effectively reweighted sensory input, and hypothesized that this alteration would result in a life-long alteration to inter-areal boundary position, topographical connections, dLGN and V1 size and possibly gene expression (although no study had examined end-of-life gene expression prior, thus controls levels of expression were unknown). Experimental evidence reported here supports these hypotheses, and provides a novel insight into the role of extrinsic sensory input in generating neuroanatomical and genetic characteristics in mice at late adulthood.

### Effects of Long-Term Enucleation on the dLGN, Primary Visual Cortex and Barrels

The thalamus of enucleated mice exhibited a reduction of the dLGN, as revealed by CO histochemical staining of coronal sections through these structures (Figure 2). Our results confirm those published in previous reports at earlier developmental timepoints, and in different model systems (Heumann and Rabinowicz, 1980; Warton et al., 1988; Asanuma and Stanfield, 1990; Dehay et al., 1996; Izraeli et al., 2002; Williams et al., 2002; Cang et al., 2005; Dye et al., 2012). Interestingly, following 18 months of post-enucleation development and maturation, the dLGN remains small but present. The persistence of the dLGN can most likely be attributed to the timing of enucleation; though retinal input was ablated immediately following birth, it is likely that prenatal cues were sufficient in establishing the rudimentary structure, which carried on throughout life. Specifically, the thalamocortical and corticothalamic handshake between visual cortex and the LGN takes place prenatally in the mouse, and although enucleation can result in cell death in the LGN, the thalamocortical connections are maintained and likely provide intrinsic activity to the nuclei (López-Bendito and Molnár, 2003). It is interesting that the cell packing density was not significantly different in the dLGN between enucleates and controls although apoptosis is the most plausible explanation for reduced size (Figure 3).

Further analysis of CO data revealed an apparent repatterning of V1 of 18 month old enucleated animals when compared to controls (Figure 2). Loss of layer-specific gradients of CO activity, and a decrease in overall CO staining within the reduced region of enucleated V1 suggests a reduction in overall neuronal activity (Wong-Riley, 1989), particularly in layers II-IV, although it may also reflect the lower cell packing density shown in Figure 3. The persistence of V1 despite life-long absence of driven visual sensory input indicates retinotopic mapping of putative visual cortex before birth, mediated by cholinergic retinal wave activity, and patterned gene expression is sufficient to establish primary visual area, with visual input after eye opening contributing primarily to maintenance of boundaries.

Studies involving experimental manipulations of peripheral input and in congenitally blind humans point out the incredible ability of the neocortex to adapt to external perturbations. Specifically, congenitally blind individuals have shown to have a shorter detection time for auditory discrimination tasks and faster language processing when compared to their sighted counterparts (Röder et al., 1999, 2000). Neuroimaging studies of blind individuals have also demonstrated that auditory localization tasks and Braille reading activate regions normally involved in visual processing (Sadato et al., 1996; Büchel et al., 1998; Weeks et al., 2000). Furthermore, electrophysiological experiments revealed cross-modal neocortical plasticity in the *Monodelphis domestica*, whereby modifying peripheral activity via enucleation in early development resulted in an expansion of auditory and somatosensory cortical areas into what would normally be visual cortical regions in the adult cortex (Kahn and Krubitzer, 2002; Karlen et al., 2006). Our results are consistent with these studies in that bilateral enucleation at birth not only significantly reduced the size of the visual cortex in senescence, but also resulted in a significant increase of somatosensory barrel size in 18 month old enucleated animals (Figure 4), suggesting that the organization of cortical areas and amount of cortical space devoted to a particular sensory system is determined by peripheral innervation and activity patterns generated with use.

### Gene Expression Patterns Following Bilateral Enucleation

The five genes investigated here have been previously implicated in early neocortical development (Suzuki et al., 1997; Mackarehtschian et al., 1999; Miyashita-Lin et al., 1999; Vanderhaeghen et al., 2000; Grove and Fukuchi-Shimogori, 2003; Nakagawa and O’Leary, 2003; Chou et al., 2009; Tomassy et al., 2010), and demonstrate a strong early onset coinciding with critical periods followed by a neocortical expression profile that declines with age. The presence of key developmental genes during late adulthood, particularly in the neocortex and white matter tracts (Figure 5), regardless of early sensory deprivation, suggests a possible secondary function in senescence. Our findings are reminiscent of those uncovered in microarray, genetic and biochemical studies of the mouse transcriptome during senescence. Aging is accompanied by expression changes in numerous genes, as well as unique molecular, cellular and morphological modifications, and these transitions are capable of masking some manipulations to the model organism (Kedmi and Orr-Urtreger, 2007; Yankner et al., 2008; Oh et al., 2011). Key transcriptional changes in aging occur to genes involved in the synaptic calcium signaling system including calcineurin Bα, calmodulin 1 and 3, CAM kinase IIα and IV, and multiple protein kinase C isoforms, accompanying alterations to ribosome biogenesis and immune response genes, which likely mediate many of these age-related changes (Kedmi and Orr-Urtreger, 2007; Yankner et al., 2008). Animals enucleated at birth displayed significantly reduced expression of secretogranin II (ScgII), a member of the chromogranin family, in the visual cortex of young adults (Paulussen et al., 2011). As ScgII contributes to neurite outgrowth and neural plasticity via the modulation of a range of neuropeptides and neurotransmitters throughout the lifespan of mice, action of this molecule provides a possible mechanism by which visual cortex plasticity can be maintained (Fischer-Colbrie et al., 1995, 2005; Shyu et al., 2008; Paulussen et al., 2011).

### INC Alterations in Response to Early Bilateral Enucleation and the Correlation with Changes in Cortical Gene Expression

Somatosensory and visual cortex INC development progresses along a well documented trajectory, with well-defined areal boundaries established by P10 carrying on through adulthood (Dye et al., 2011a,b). In our previous investigation of P10 mice enucleated at birth, we reported altered patterning at areal boundaries, with reduced refinement apparent at the medial S-V border (Dye et al., 2012). As this study was conducted prior to natural eye opening, it is possible that the alterations observed were driven by the interruption of spontaneous retinal wave activity and trans-eyelid derived visual sensory information (Meister et al., 1991; Krug et al., 2001; Cang et al., 2005; Mrsic-Flogel et al., 2005). Here we demonstrate a more pronounced phenotype of the same ectopic connectivity at the medial S-V border where somatosensory connections have expanded into regions of extrastriate cortex (Figures 6, 7). While a more defined shift of INCs is present at the S-V border, it is important to note that the overall rostral to caudal extent of connections associated with either somatosensory or visual DPLs remains relatively stable across both groups, despite a lifetime of ablated retinal input (Figure 6). This striking similarity suggests that the spontaneous retinal activity and intrinsic neocortical gene expression that generates the critical aspects of early developmental patterning are sufficient for the lifelong patterning of the majority of INCs. The lack of differences in overall cortical projections coupled with inaccurate targeting at the S-V border found here is consistent with work by Laing et al. (2012) who found that striate-extrastriate projections progress through similar developmental phases in both control and enucleated rats, with each group generating small, dynamic filopodium-like branches on area 17 and 18A axons despite lack of topographical precision in enucleates. The inaccurate mapping of INCs observed in the present study could be a consequence of the early loss of retinal topographic cues, resulting in guidance generated solely from intrinsic cues. Indeed, *ephrin A5* transcript patterns in the early postnatal neocortex are altered in enucleated mice, and are correlated with shifts in connectivity (Dye et al., 2012). Furthermore it has been long accepted that *ephrin A5* activity mediates a number of features of axonal growth and pathfinding including topography-specific axonal branching, and the proper expression of these molecules; thus, correct targeting is only achieved under normal developmental patterns of spontaneous activity (Dütting et al., 1999; Yates et al., 2001; Hanson and Landmesser, 2004; Huberman et al., 2005). Studies of callosal projection patterns reveal a similar developmental paradigm where eyes are only required briefly at the initiation of pathway formation, but not for the entirety of development (Olavarria et al., 1987; Olavarria and Hiroi, 2003).

Through a coupled analysis of rostromedial gene expression and connections demonstrating the S-V area border, we observed a noted disorganization of the border in the enucleated animals in a region of greatly diminished gene expression of *RZRβ, COUP-TFI*, and *ephrin A5* (Figure 8). This observation supports the idea that regions of cortical gene expression, specifically graded or patterned with a clear boundary, play a role in both area border development early on and border maintenance throughout life. In the life-long visually deprived animal, cortical plasticity mechanisms diminish the presence of visual cortex, thus altering the normal features and location of the S-V border. This is represented in our data through an analysis of INCs. How this region co-registers with an atypical absence of gene expression, which is normally present at the border in sighted animals, is a novel and exciting finding.

## Conclusion

Bilateral enucleation at birth drives a persistent, lifelong reduction to the size of LGN in the dorsal thalamus and the V1, and modifies axonal projections at sensory area borders. We hypothesize that following initial patterning of the neocortex, early neocortical gene expression, along with sensory input, may function to guide normal areal boundary formation. The observation that cKO driven elimination of nearly all TCAs from the initiation of their development caused altered areal gene expression patterns, particularly within V1 and S1 where inter-areal boundary definition was lost (Vue et al., 2013), combined with the finding that altered *ephrin A5* expression patterns correlated with aberrant INCs at areal boundaries (Dye et al., 2012), demonstrates the critical role of TCA input and proper gene expression in the establishment of neocortical areas. Interestingly, although gene expression levels involved in neocortical areal boundary establishment examined here are still present in late adulthood, albeit greatly reduced as a result of early sensory deprivation, it is also possible that other molecules, such as those guided by ScgII expression maintain visual system plasticity in senescence. Future studies that provide transcriptome-wide analyses in late adulthood, such as microarray or RNA-Seq analysis, are needed to further our investigation into how activity-dependent mechanisms, such as sensory input and intrinsic mechanisms such as gene expression interact to maintain areal features and sensory plasticity during aging.

## Conflict of Interest Statement

The authors declare that the research was conducted in the absence of any commercial or financial relationships that could be construed as a potential conflict of interest.
